# Feasibility study evaluating arrhythmogenesis and cardiac damage after heart‐base irradiation in mice: A brief communication

**DOI:** 10.1002/vms3.303

**Published:** 2020-06-10

**Authors:** James Elliott, Keith Linder, Michael W. Nolan

**Affiliations:** ^1^ Department of Clinical Sciences College of Veterinary Medicine North Carolina State University Raleigh NC USA; ^2^ Department of Population Health and Population Biology College of Veterinary Medicine North Carolina State University Raleigh NC USA; ^3^ Comparative Medicine Institute North Carolina State University Raleigh NC USA

**Keywords:** animal models, arrhythmia, radiation, radiobiology, radiotherapy

## Abstract

Radiation‐induced heart disease (RIHD) is a potential cause of morbidity and mortality in dogs undergoing thoracic irradiation. Arrhythmias and sudden death have been documented in dogs undergoing stereotactic body radiation therapy for heart base tumours. A study was proposed to interrogate the effect of different stereotactic‐like radiation prescriptions on RIHD development, including arrhythmogenesis and classical histological endpoints in a mouse model. A pilot study was performed initially. The heart base of CD1 (*n* = 3) and C57Bl/6J (*n* = 3) female mice were irradiated (12 Gy × 3, daily) with a clinical linear accelerator. No significant adverse effects were noted and each mouse survived the entire subsequent 3‐month observation period. At various time points, no arrhythmias were identified on ECG analysis. Cardiac histology (haematoxylin and eosin, and picrosirius red staining) was performed at 3 months. In a single CD1 mouse and two C57BI/6J mice, multifocal, minimal, peri‐vascular lymphoplasmacytic inflammation was noted within the irradiated proximal heart. In one mouse of each strain, a small, single focus of fibrinoid vascular necrosis was observed. Overall, there was no significant myocardial necrosis, atrophy or inflammation. Picrosirius red staining revealed no evidence of fibrosis in any mouse. Dosimetric verification indicated that the irradiation was successful and delivered as planned, with an average predicted‐to‐measured dose‐difference within 5%. While this study did not demonstrate significant arrhythmogenesis, certain modifications of the experimental mouse irradiation procedures are discussed which may enable more translationally relevant modelling of the canine cardiac response to SBRT‐like irradiation.

## Graphical abstract

This article summarises the results of a feasibility study evaluating arrhythmogenesis and cardiac damage after heart‐base irradiation in mice. After delivery of a severely hypofractionated radiation protocol to the proximal heart of mice, no arrhythmogenesis was elicited and minimal histologic change was found. Further modifications of this technique are required before widespread utility to study radiation‐induced arrhythmogenesis and cardiac damage in a mouse model.

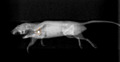

## 1 INTRODUCTION

Radiation response of the heart is well‐described in dogs, including changes in cardiac structure, function and conduction (Gavin & Gillette, 1982; Gillette & McChesney, 1986; Gillette, McChesney, & Hoopes, 1985; Gillette et al., 1992; McChesney, Gillette, & Powers, 1988; Phillips, Reid, & Rugh, 1964; Senderoff, Kahn, Peck, & Baronofsky, 1960; Senderoff, Kaneko, Beck, & Baronofsky, 1961; Takaoka, 1969). However, radiation‐induced arrhythmogenesis has often been ignored as ‘subclinical’. This dogma is challenged by a recent study of stereotactic body radiation therapy in canine heart base tumours, which revealed a high frequency of clinically significant arrhythmias in the first 6 months following therapy (Magestro, Gieger, & Nolan, 2018). Reducing the incidence of such arrhythmias may improve survival. Achieving that goal is made difficult by uncertainty as to whether these arrhythmias resulted from cardiac irradiation, or the mere presence of an arrhythmogenic cardiac tumour. Confirming the aetiology in pet dogs is not straightforward. Thus, we sought to develop an animal model of heart base irradiation, in which we could evaluate the impact of radiation dose and fractionation on the risk of RIHD. We also envisioned future use of this model to evaluate putative radiomitigators (e.g. via calculation of dose‐modifying factors). As most animal model studies have focussed on the effects of whole heart irradiation, proximal heart irradiation was deemed more translatable to clinical veterinary practice. The overarching aim was to provide preliminary data prior to companion animal clinical trials.

This manuscript describes the methodology and results of a pilot study that was undertaken using the highest radiation dose prescription planned for use in a subsequent larger study that would rigorously define the dose–response relationship for proximal heart irradiation in mice. The goals were to confirm that a radiation treatment plan was deliverable to a very small target with the available radiation equipment, to ensure there would be no rapid mortality or peracute pathology that could invalidate the study, and to confirm that we could induce scorable, classic, histological radiation‐induced heart base damage. Additionally, we aimed to determine the most appropriate mouse strain to study and assess the timing of arrhythmogenesis and other study endpoints with respect to the irradiation procedure.

## 2 METHODS

Female, young‐adult, 23–27 g C57Bl/6J (*n* = 3) and CD1 (*n* = 3) mice were acquired from a commercial vendor (The Jackson Laboratory). After a 2‐week acclimatization, mice underwent partial cardiac irradiation (PCI) performed with a linear accelerator (Varian Novalis TX, Varian Medical Systems, Inc) using 6 MV photons. Two strains were used, representing differential sensitivity to developing pleural effusion and/or pulmonary fibrosis post irradiation (Walkin et al., 2013). All animal procedures were performed in accordance with the Guidelines for Care and Use of Laboratory Animals (2011) and experiments were approved by the Institutional Animal Care and Use Committee (IACUC).

**FIGURE 1:**
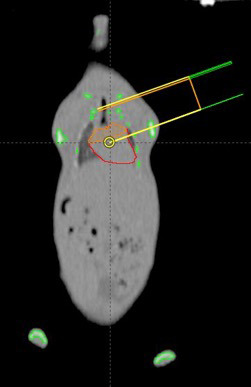
A computerized radiation treatment plan was generated, using an asymmetric collimator opening (half‐beam block) and a single, tangential beam to irradiate the heart base of the study mice. The tissue outlined in red is the heart; the orange contour represents the targeted "heart base"

**FIGURE 2:**
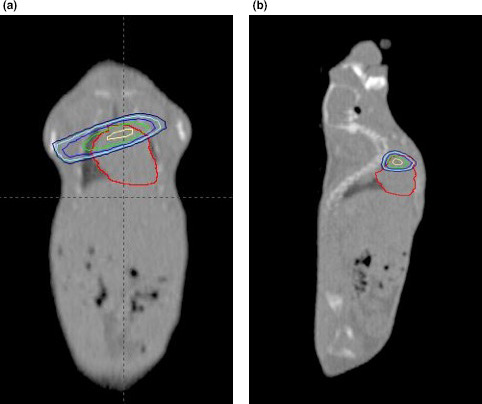
Isodose lines, in two planes, generated on radiation treatment planning software for irradiation of the heart base of a mouse. The outermost line represents the 80% isodose line. (a) A coronal section of a non‐contrast CT scan of an entire mouse; the top of the image is the thoracic inlet and the bottom of the image is the caudal abdomen. (b) A sagittal section of the same mouse and CT scan. As in Figure 1, the red contour identifies the cardiac silhouette and the orange contour represents the targeted/irradiated "heart‐base"

**FIGURE 3:**
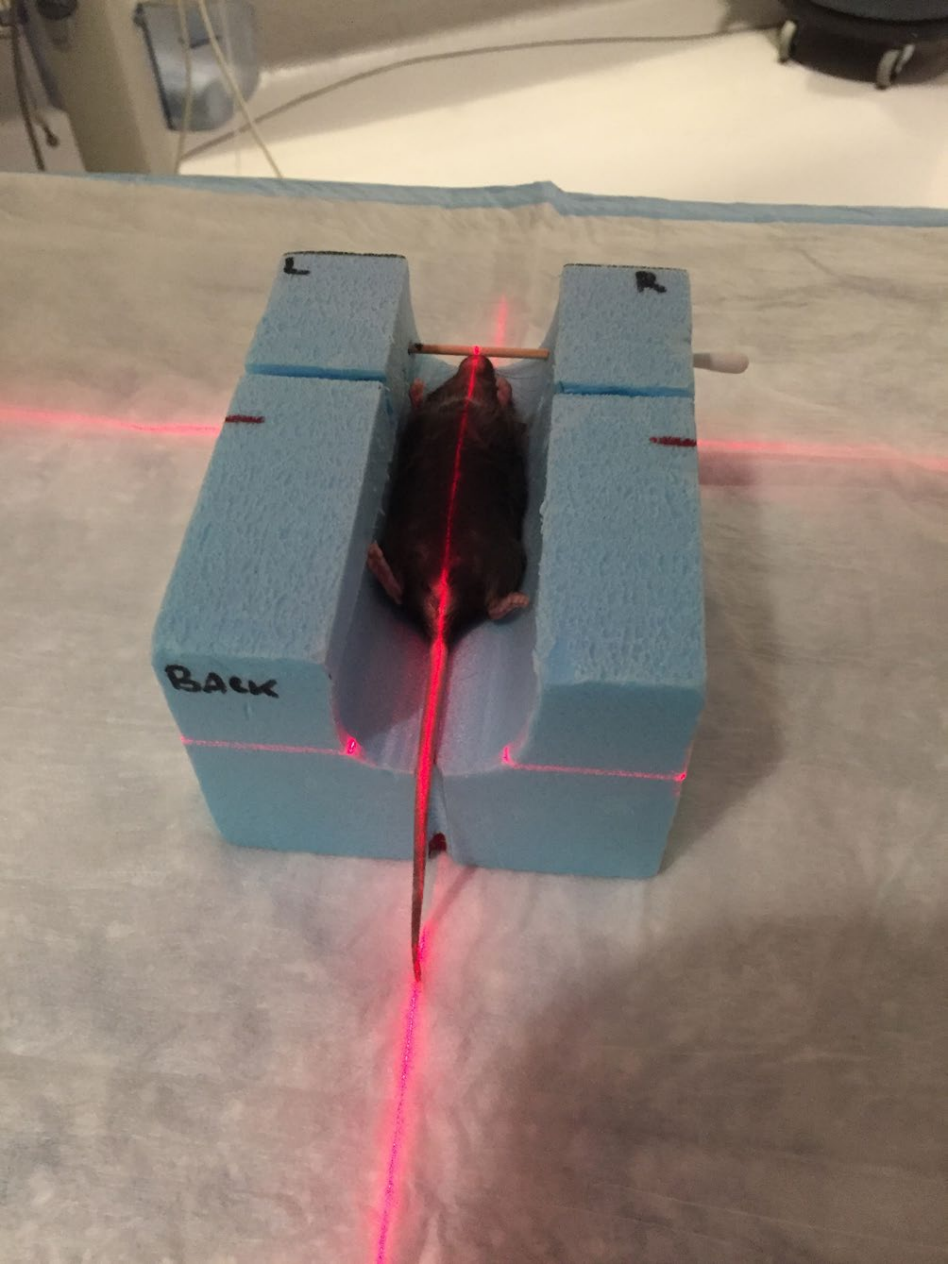
The custom‐built polystyrene immobilization device utilized for the planning CT scan and the irradiation of all mice

Computed tomography (CT) was performed on a single mouse, under anaesthesia. A computerized treatment plan (Eclipse™ version 11.0.47, Varian) was created that could be applied to all similarly sized mice. The proximal third of the heart (i.e. the heart base) was irradiated, sparing as much lung tissue as feasible by means of a 2.5‐mm multi‐leaf collimator. The proximal heart structure was contoured so as to confidently include both atria. The prescription was 12 Gy × 3, daily fractions. The treatment isocentre was placed at the edge of the proximal heart contour; this allowed asymmetric closing of the primary collimator (half‐beam block) to reduce penumbra and minimize dose to the ventral heart. For the same reason, a single, tangential beam was used (Figure 1). The plan was normalized so that 95% of the planned target volume was covered by 95% of the prescription (Figure 2). For irradiation, mice were anaesthetized with 2%–3% isoflurane (Isoflurane, USP, Piramal Ltd) and 2 L per minute oxygen (box induction and subsequently maintained via face mask) and positioned in a custom‐built positioning device, which was also used for the planning CT. This was a polystyrene trough with a wooden marker to align to the tip of the nose, and mice were positioned in dorsal recumbency (Figure 3). Orthogonal kilovoltage (kV) radiographs were made (using the linear accelerator's on‐board imaging system; OBI) on each mouse immediately prior to treatment to allow anatomic matching, based on the cardiac silhouette.

**FIGURE 4:**
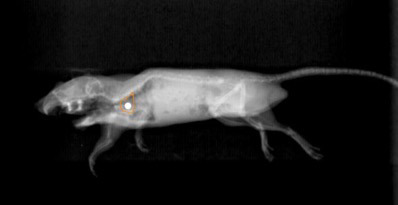
A kilovoltage radiograph of a cadaver mouse in the treatment position, which has had a thermoluminescent dosimeter (attached to a radiopaque marker) inserted to the level of heart base. The proximal heart contour, derived from digitally reconstructed radiographs, is shown as an orange line

To verify accurate targeting and dosimetry, five freshly killed mice of similar size to the test mice were irradiated following implantation with thermoluminescent dosimeters (TLDs) at the heart base (placed via mini thoracotomy). A radiopaque fiducial was attached to the TLD, enabling TLD position to be radiographically verified prior to irradiation (Figure 4). Cadaver was treated with 500 cGy, using the treatment plan described above, with appropriately scaled monitor units. TLD analysis was performed by the University of Wisconsin‐Madison, Radiation Calibration Laboratory, WI, USA.

**FIGURE 5:**
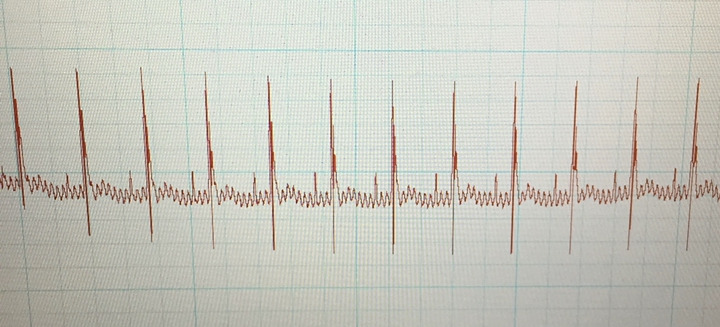
An ECG recording from a CD‐1 mouse, 4 weeks post‐irradiation. Note the presence of visible p‐waves, narrow QRS complexes and undulating baseline

Electrocardiogram (ECG) (lead II via limb electrodes, 50 mm/s speed, under brief isoflurane anaesthesia) readings were acquired prior to irradiation (baseline) and weekly‐to‐fortnightly thereafter for 3 months. Readings were acquired following a 5‐min acclimatization to a light plane of isoflurane anaesthesia. Needle electrodes (30‐gauge) were used; electrocardiograms were recorded (PowerLab data acquisition system model ML866, ADInstruments; and Animal Bio Amp model ML136, ADInstruments). Settings were as follows: range (10–20 mV), sampling rate (4 kHz), high‐pass filter (1 Hz), low‐pass filter (1 kHz) and absence of Notch filter and with all electrical equipment as far away from the setup as practicable. At least 60 s of signal was recorded, with the tracing visually reviewed for diagnostic utility and for detecting arrhythmias and aberrant ECG complexes (Figure 5). For this pilot study, the parameters assessed were average heart rate during each analysis and the traces were visually inspected for aberrant complexes or rhythms.

Mice were checked daily for signs of distress, abnormal nesting behaviour (subjectively evaluated) or radiation adverse effects; they were weighed weekly.

At the end of the 3‐month study period, mice were killed (carbon dioxide and cervical dislocation) and hearts with heart base tissue were fixed in 10% neutral‐buffered formalin. Hearts were sectioned on the longitudinal axis through the heart base tissue and great vessels. Tissues were routinely processed for histology and embedded in paraffin wax, sectioned to approximately 5 µm, and stained with haematoxylin and eosin (H&E) or picrosirius red for fibrosis. Two whole heart sections were evaluated per mouse and included the heart base tissue, right and left atria, right and left ventricles and the interventricular septum. Histological lesions, including cardiomyocyte degeneration, necrosis, fibrosis, inflammation and vascular damage, were evaluated by a veterinary pathologist (K.L.).

## 3 RESULTS

Estimated lung dose was assessed by means of a dose–volume histogram and volumes of the lung receiving 90%, 50% and 20% of the total prescribed dose were 2.6%, 18.4% and 40%, respectively.

TLD measurements made in mouse cadavers, and using the same clinical linear accelerator as for mice irradiation, indicate that the dose delivered was commensurate with the intended prescription dose (Table 1).

**TABLE 1:** Table of thermoluminescent dosimeter (TLD) data for irradiated mouse cadavers

Thermoluminescent dosimeter data	
Number of TLDs	*n* = 5
Range	338–551 cGy
Mean dose	480 cGy
Standard deviation	87 cGy
Standard error	40 cGy
95% confidence interval (of the mean)	372–587 cGy
Difference between calculated and measured	−4.1%

In live mice, the cardiac silhouette and body contour were easily visualized on OBI‐generated orthogonal kV radiographs; this allowed accurate matching to digitally reconstructed radiographs (DRR) that had been generated from the radiation treatment plan.

All six mice survived the entire 3‐month observation period. There were no observed immediate adverse effects, with rapid return to normal eating, grooming and nesting behaviour. No constitutional adverse effects were observed, and specifically there was no evidence of tachypnoea or dyspnoea. All mice continued to gain weight until the time of euthanasia. Only mild adverse effects of radiation were noted. Alopecia, without dermatitis, was observed in the right axillae of all six mice after 4 weeks, at the site of beam exit. Hair regrowth began 4 weeks after radiation treatment in the white, CD1 mice, but remained sparse at euthanasia. In the pigmented (black) C57Bl/6J mice, leucotrichia developed after 4 weeks.

During the study period, there was no significant change in heart rate in any of the mice (Table 2) and no aberrant complexes or rhythms were noted at any time period.

**TABLE 2:** Table of the heart rate of six mice at various time points following proximal heart base irradiation. Week 0 is baseline (prior to irradiation)

Mouse strain/stock	Subject	Weeks post irradiation								
		0	1	2	3	5	7	9	10	12
		Heart rate (beats per minute)								
CD‐1	1	366	342	360	366	342	348	342	354	342
	2	342	366	384	342	378	390	342	330	366
	3	324	324	324	348	360	366	378	324	348
	*p*‐value	—	>0.9999	0.9903	0.9994	0.9455	0.6644	0.9971	0.9994	0.9994
C57Bl/6J	1	348	378	330	336	330	360	360	324	330
	2	348	336	360	318	348	378	306	324	336
	3	378	378	354	300	330	354	330	348	354
	*p*‐value	—	>0.9999	0.9971	0.1049	0.7552	>0.9999	0.5690	0.5690	0.8992

**FIGURE 6:**
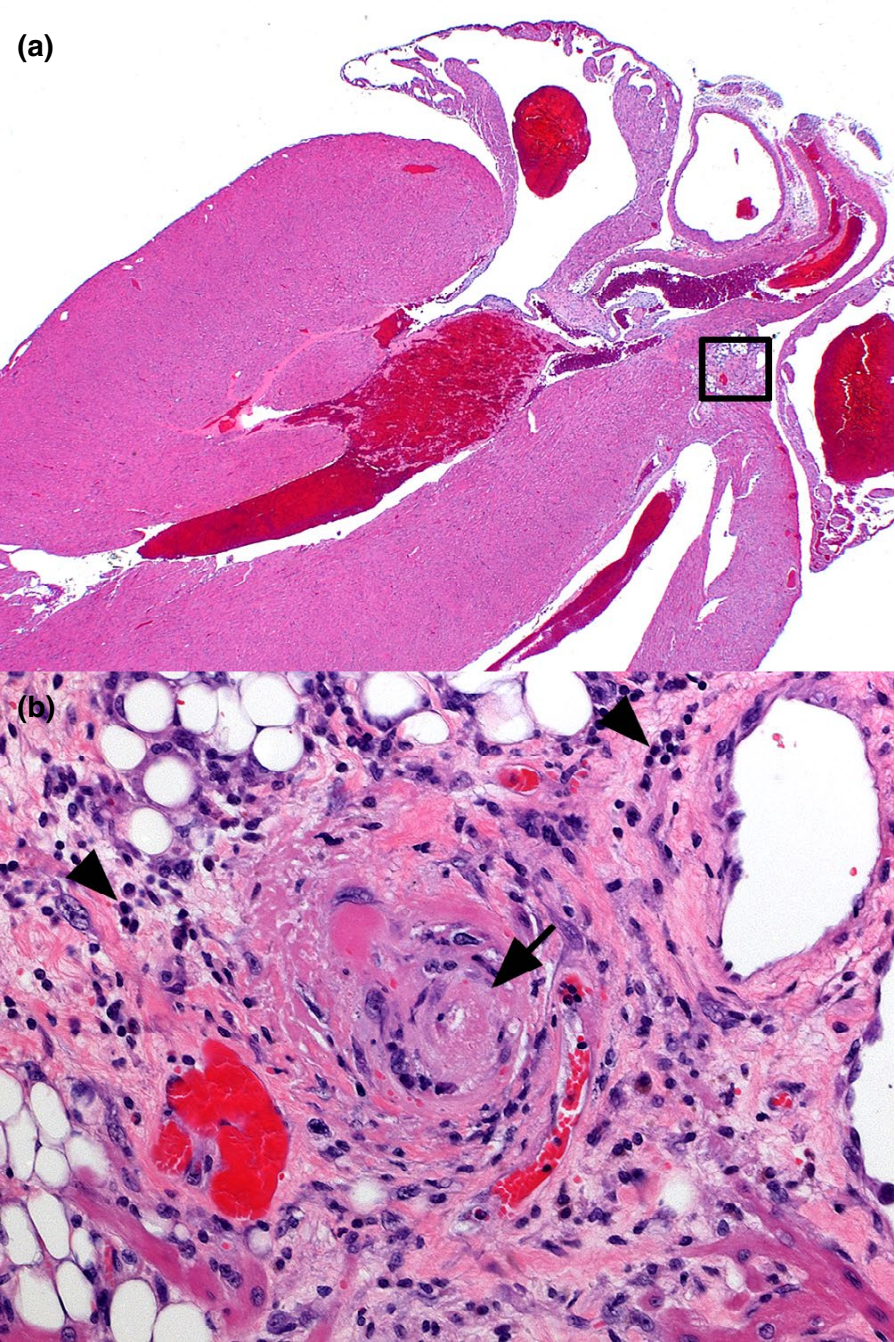
CD‐1 Mouse, irradiated heart. (a) Longitudinal axis section includes all heart chambers and heart base. There is no significant radiation‐induced necrosis, fibrosis or atrophy of the myocardium. 5× magnification. (b) Inset from image (a). One small blood vessel (arrow) is partly obscured by fibrinoid vascular necrosis. Minimal to mild perivascular infiltration includes lymphocytes and plasma cells (arrowheads). 20× magnification. Haematoxylin and eosin

On gross post‐mortem examination, there were no macroscopic pathological changes to the heart or other thoracic organs. Specifically, there were no pericardial or pleural effusions evident. The lung tissue surrounding the heart was visibly unremarkable. In a single CD1 mouse and two C57B1/6J mice, minimal to mild, multifocal, peri‐vascular lymphoplasmacytic inflammation was noted in the proximal heart and heart base area. In one mouse of each strain, a small, single focus of fibrinoid vascular necrosis was observed within the heart base area close to the epicardium (Figure 6). Overall, inflammation was considered insignificant. There was no myocardial degeneration, necrosis or atrophy. Neither H&E nor picrosirius red staining revealed any evidence of myocardial fibrosis in any mouse.

## 4 DISCUSSION

The present pilot study demonstrates that a clinical linear accelerator can be used to precisely and accurately perform experimental heart base irradiations in mice. Despite various dosimetry challenges in small fields for radiotherapy (Andreo, 2018), TLD‐based dosimetric verifications indicated an average predicted‐to‐measured dose‐difference within 5%. Nonetheless, the current methodologies resulted in neither arrhythmogenesis nor classic histological changes. With little serious/scorable histological change and without gross arrhythmia by 3 months post irradiation, we opted not to move to a larger study using these methods. Thus, the purpose of this communication is to report our methodology and discuss its limitations, enabling future researchers to benefit from our experiences and consider model modifications.

Extrapolating from previously published studies (Gavin & Gillette, 1982; Hu et al., 2009; Schultz‐Hector, 1992; Seemann et al., 2012; Zhang et al., 2015), it seems reasonable to have expected arrhythmia and histological change to have been common at this radiation dose level (thus justifying our sample size for this feasibility study). However, we cannot entirely exclude the possibility that small sample size alone explains our failure to detect expected changes in conduction and morphology.

The α/β ratio (a radiobiological term that can be used to predict the impact of dose per fraction and/or total dose of radiation on risk of radiotoxicity) remains undefined for most cardiac arrhythmias. However, estimated α/β values for canine cardiac toxicoses are 2.5 Gy for pericardial fibrosis, 2.7 Gy for heart rates exceeding 135 beats per minute, 3.2 Gy for myocytolysis and 5.4 Gy for ST segment changes (Gavin & Gillette, 1982). These values are typical of late responding tissues and historic studies suggest that 3 months should have been adequate follow‐up for documentation of cardiac late effects in our mouse model. However, it is possible that longer follow‐up would have revealed substantial changes that we missed solely due to a short study period (Gillette et al., 1985; Gillette, Powers, Orton, & Gillette, 1991). Additionally, radiation prescriptions with a higher biologically effective dose may be more successful in inducing cardiac injury; this may, however, be less translatable to dogs, as the dose would be in far excess of that which is utilized clinically.

Radiation‐induced arrhythmias can develop as subacute and late effects of radiation; the underlying cause for radiation‐induced arrhythmogenesis likely varies with the type and time to onset. Based on time to development of arrhythmias in canine patients having undergone SBRT for HBT, we were most interested in subacute arrhythmias. Thus, the focus of this pilot study was to determine whether partial heart irradiation induces arrhythmias within 3–4 months in mice. We were not interested in studying arrhythmias that arise later, and that is what drove the design of a relatively short experiment. While no arrhythmias were detected, it was not possible to accurately measure and record the P‐R interval, QRS interval or S‐T elevation, due to undulation in the ECG baseline (baseline noise). It is therefore possible that subtle conduction disturbances were unidentified. More sensitive ECG analysis may ascertain if there is a dose–response relationship with respect to rhythm disturbance development, or severity. Additionally, arrhythmias could have been missed due to intermittent ECG analysis. To address this limitation, telemetry with continuously measured and recorded ECG could be considered (McCauley & Wehrens, 2010). The ECGs in this study were performed in anaesthetized mice; though anaesthesia is known to be arrhythmogenic, this is unlikely to have been an issue, given the lack of demonstrable dysrhythmia (Atlee, 1985). Conscious ECG via telemetry would also address this issue in future investigations. The severity of radiation effects can also depend on the volume of tissue irradiated, therefore it is possible that no arrhythmias were seen due to the small volume of heart irradiation (i.e. the whole heart was not irradiated, as in most of the previous cited studies. However, partial heart irradiation was mandatory in this study, given that the goal was to recapitulate the type of treatments performed in highly conformal, severely hypofractionated, SBRT‐like protocols in companion animals. It is also important to consider that the lack of demonstrable arrhythmia in this study could serve as provisional evidence that this type of SBRT‐like heart base irradiation is not arrhythmogenic, and that the dysrhythmias seen in a previous canine study were due to tumour infiltration causing conduction disturbance, as opposed to RIHD.

Another consideration is that differences in mouse strain likely influence the type of RIHD that develops in mice. We chose to evaluate CD1 and C57Bl/6J mice. CD1 mice are significantly more cost‐effective, however, the potential advantages of the C57Bl/6J strain were decreased sensitivity to radiation‐induced lung injury and pleural effusion (Dabjan et al., 2016; Walkin et al., 2013), both of which could have compromised the study due to early death or severe morbidity. Given the lack of problematic lung injury, CD1 mice could be considered for future studies, if utilizing similar methodology to this study. The authors are not aware of any reports describing the spontaneous occurrence of arrhythmias in mice, the intrinsic sensitivity of various mouse strains/stock to developing arrhythmias has been studied in the context of induced arrhythmia models. Following stimulation (beta adrenergic activation), C57Bl/6 mice have an intermediate susceptibility to ventricular arrhythmias versus Balb/c (most susceptible), and both FVB and Black Swiss mice (least susceptible; Jelinek, Wallach, Ehmke, & Schwoerer, 2018). That said, others have reported that electrical stimulation of the heart more readily induces ventricular tachycardias in FVB and Black Swiss mice, versus C57Bl/6 mice (Maguire et al., 2003). Thus, the influence of genetic background on susceptibility seems to vary with the underlying cause for arrhythmogenesis. Nonetheless, based on these published data, future consideration of mice as a model for radiation arrhythmogenesis following partial heart irradiation should likely include evaluation of Balb/c mice.

There is also evidence that sex can influence the risk of cardiopulmonary injury (Jackson et al., 2017; Stojkovic et al., 2016). Females appear to be more sensitive to thoracic irradiation, with increased death due to lung disease and increased pleural effusion (Jackson et al., 2017). While there appears to be little data pertaining to RIHD, it seems prudent for sex to be a consideration in any future investigations.

Concurrent lung irradiation can potentiate RIHD (Sievert, Stangl, Steiger, & Multhoff, 2018), and efforts should be made to minimize the volume of lung tissue in the treatment field. In this study, this was achieved with a single tangential beam and the use of a micro‐MLC to block non‐target tissues. Dose–volume histogram analysis was deemed acceptable by the investigators. While we cannot comment on the effect of concurrent lung irradiation on the effects of the hearts of mice in this study, the lack of observed breathing changes in the mice post irradiation and lack of effusions suggest tolerable lung irradiation was performed. Ultimately, the result of inclusion of significant lung (or other organ) volumes would be to exacerbate cardiac pathology. As we ultimately saw little pathology, this is unlikely to have affected the results described herein. Despite this, the creation of small and conformal irradiation fields is preferable, and can be challenging with clinical linear accelerators, as used in this study. Small animal kilovoltage (kV) irradiators have a dramatically smaller penumbra, with less scatter outside the radiation field, and can therefore produce highly conformal dose distributions that may be better suited for cardiac substructure irradiation (Sievert et al., 2018). Limited study of purpose‐bred dogs could also enable accurate targeting, and results which are directly translatable to pet dogs. Microphysiological approaches, such as cardiac organoids and ‘heart‐on‐a‐chip’ devices (Zhang & Yu, 2016) are capable of recapitulating some of the physiology of the beating heart, and in the future, could become an alternative approach to the study of RIHD, including arrhythmogenesis.

The positioning device used was effective, and resulted in subjectively few cranio‐caudal and lateral shifts to isocentre being required, following pretreatment imaging. Improvements could include a more conforming device to prevent patient roll, which was documented in some patients and required repositioning and re‐imaging prior to treatment, which prolonged anaesthesia time. Minimizing shifts required is advantageous, as any radiation couch shifts cause some jerking of the table which could result in subject movement between imaging and treatment. Couch movements also have a finite accuracy. These issues could result in either over‐ or underdosing of the target, particularly with highly conformal irradiation techniques. For this reason, it could be considered to re‐image subjects in the treatment position after couch shifts have been applied, to ensure there was no inadvertent patient motion. It is possible that given the small field size, kV cone‐beam CT could have provided a more accurate verification of patient positioning. This was not considered a significant limitation given the validation in dose deposition provided by the TLD data. Orthogonal kilovoltage radiographs appeared to be a valid imaging method for verifying treatment position in this study.

Due to their small size, TLDs are convenient for point dose measurements in phantoms as well as for in vivo dosimetry during radiotherapy treatment. The thermoluminescent response is not linear for large doses. At high doses, above 10 Gy, it exhibits a supralinear behaviour; this supralinearity has been one of the main drawbacks to their use in high‐dose dosimetry (Liuzzi et al., 2020; Liuzzi, Savino, D'Avino, Pugliese, & Cella, 2015). For this reason, a smaller dose was given (with scaled monitor units from the original treatment plan) via the same treatment machine.

As can be seen from the radiograph in Figure 4, the mandible of the cadaver mouse for TLD analysis was dissected to allow forceps to insert the TLD chip towards the heart base. It is possible that this dissection could affect radiation dosimetry, by disruption of tissue planes and introduction of air, which could result in a loss of electronic equilibrium and lack of dose build‐up. TLD placement was performed as carefully as possible and tangential beam orientation (Figure 1) meant that the radiation beam is unlikely to have passed through the proximally disrupted tissues. Ultimately, accuracy of the delivered dose was confirmed.

For such precise local heart irradiation as discussed herein, it is important to consider the potential effects of respiratory and cardiac motion on the absorbed dose in the proximal heart. Such physiological motion can cause movement of the radiation target (i.e. the proximal heart) and is typically accounted for in radiation treatment plans by irradiation of an extra margin of tissue around the target, known as an internal target volume (ITV). This avoids underdosing of the target, which could happen if it moved outside the treatment field during radiation delivery. While such an additional margin was not specifically added to the proximal heart contour in this study, the degree of physiological movement of the proximal heart was anticipated to be very low in mice, based on previous experience of the authors. This was confirmed by fluoroscopic imaging of the first mouse irradiated while in the treatment position, using the linear accelerator's on‐board kilovoltage imaging system. Regardless, it cannot be completely discounted that physiological movement could have underdosed part of the target in this study, and a small ITV could be considered for future studies using similar methodology.

The use of three animals per group is too few to draw meaningful global conclusions, and there are no controls, so the effects of irradiation (as opposed to other factors such as age, procedures, anaesthesia, positioning, ECG, time and background disease cannot be assessed. However, the goal of this manuscript was to report preliminary findings only and future work would mandate the study of a significantly larger number of subjects.

Larger studies in the future should also include standard histological assessment of other organs at risk which could affect cardiac response to irradiation, such as the lungs.

In conclusion, the described method of irradiating a small murine target with a clinical linear accelerator was both successful and accurate. While this study did not demonstrate significant arrhythmogenesis, or expected classical tissue endpoints of radiation, it does provide a basis for modification of this model to further study the cardiac response to SBRT‐like irradiation.

## AUTHOR CONTRIBUTION

**James Elliott:** Conceptualization; Data curation; Formal analysis; Funding acquisition; Investigation; Methodology; Project administration; Supervision; Validation; Writing‐original draft; Writing‐review & editing. **Michael W Nolan:** Conceptualization; Formal analysis; Funding acquisition; Investigation; Methodology; Project administration; Resources; Supervision; Validation; Writing‐original draft; Writing‐review & editing. **Keith Linder:** Conceptualization; Formal analysis; Methodology; Resources; Supervision; Writing‐original draft; Writing‐review & editing.
